# Supplementary material for: Surface Microbiome Profiling of Dental Elevators Using Third‐Generation Sequencing: Implications for Infection Control in Dental Practice
Source: Microbiologyopen. 2025 Dec 8;14(6):e70178. doi: 10.1002/mbo3.70178 (PMC12685768; doi:10.1002/mbo3.70178)
Supplement: Supplementary file 1 — customer_backup. [file MBO3-14-e70178-s001.zip › customer_backup/customer_backup/taxa_summary/krona/samples/M14.Krona.html]

Javascript must be enabled to view this page.

members
magnitude
magnitudeUnassigned

M14.krona

59664

59663

51

51

51

51

51

27

23

1

8987

70

70

70

70

70

161

161

2

2

2

27

16

1

1

14

1

1

1

1

1

1

1

1

2

1

1

1

1

4

4

132

79

63

1

15

53

53

1466

69

45

34

34

2

2

9

9

24

24

24

215

49

49

49

4

4

4

4

4

4

158

158

158

309

12

12

12

81

81

81

7

7

7

195

195

195

14

14

14

7

7

7

7

866

46

42

42

4

2

2

242

242

1

15

226

316

316

316

262

35

35

197

197

30

30

2252

1267

742

569

89

1

1

212

157

109

91

11

80

60

36

9

15

22

22

525

82

82

443

443

985

343

343

23

319

1

642

1

1

641

641

4985

93

93

1

1

92

2

2

2

14

21

20

1

30

8

8

8

8

17

7

7

7

10

10

10

10

10

10

10

60

60

9

9

51

9

7

31

4

61

61

2

2

9

6

3

50

50

499

80

3

3

1

1

72

72

2

2

2

2

419

28

11

17

366

9

1

1

132

15

13

2

15

19

3

2

62

7

5

15

48

14

3

25

25

32

32

28

1

7

20

4

4

40

40

40

1

2

1

21

13

2

270

123

38

38

83

83

2

2

30

5

5

25

25

117

14

14

19

19

84

84

40

40

16

2

6

8

14

9

1

4

6

6

4

4

21

13

13

1

2

4

6

8

3

1

2

3

3

2

2

3820

30

25

25

4

4

1

1

3318

403

403

7

7

1

1

445

445

2462

1642

10

559

251

8

8

8

313

258

258

55

55

118

2

2

17

17

10

10

5

5

23

23

49

1

1

47

12

12

7

7

7

26

2

2

24

24

14

6

6

6

8

8

8

53

53

53

53

5

48

1588

1588

1588

1588

1587

586

57

46

574

60

42

135

87

1

1

616

616

616

616

616

616

1661

1661

1661

1616

1616

1615

1

45

45

45

575

575

575

7

7

7

568

568

268

300

41

17

17

8

8

8

9

9

9

24

24

1

1

1

23

23

23

44

12

5

5

2

2

2

1

1

1

1

7

3

3

3

1

1

1

3

2

2

1

1

32

30

30

30

30

2

2

2

2

26

26

26

26

26

19

7

435

1

1

1

1

1

323

323

311

311

311

12

8

8

4

3

1

110

110

42

1

1

40

40

1

1

68

68

68

1

1

1

1

1

16

16

16

16

16

16

1620

10

10

10

10

10

36

24

24

24

24

12

12

12

12

31

31

18

18

18

13

13

13

1543

1543

1056

1056

1056

34

34

34

4

4

4

87

87

87

7

7

7

21

21

21

163

142

142

21

21

171

171

171

610

226

13

13

13

13

213

79

70

70

9

9

134

44

44

1

1

89

89

137

21

21

21

6

15

116

116

17

11

6

28

28

51

19

32

20

20

85

85

85

85

1

20

1

21

18

24

17

17

17

17

17

145

145

16

16

16

129

129

129

8

8

8

8

8

8

532

7

7

7

7

7

203

203

203

58

58

130

130

15

15

322

322

322

241

189

52

75

75

6

6

764

764

764

764

652

143

509

112

112

255

63

63

63

63

63

192

75

75

75

64

11

104

43

43

31

12

54

54

54

7

7

1

6

13

13

1

1

12

12

85

85

85

10

10

10

75

16

16

59

5

3

51

209

169

31

31

19

19

12

12

138

40

40

40

69

69

69

16

16

16

13

13

13

40

14

14

9

9

5

5

26

26

26

26

20113

12285

209

209

159

159

42

42

8

8

575

127

127

127

349

33

25

8

37

28

9

4

4

275

275

99

99

99

17

5

5

5

12

12

12

93

93

92

92

1

1

24

24

24

24

6803

6803

1

1

266

61

16

88

1

100

56

56

26

26

200

200

19

19

12

6

6

1

1

886

532

79

275

5170

6

6

469

142

31

988

8

1364

1523

139

19

452

23

166

11

6

149

34

34

34

34

2602

210

1

1

15

15

14

14

180

23

19

138

343

71

71

135

26

8

94

1

1

5

137

137

108

6

1

5

71

71

31

24

1

6

58

58

57

1

198

1

1

2

2

20

20

61

61

114

17

97

230

15

15

169

169

46

46

498

15

15

6

6

193

193

194

11

82

73

2

26

39

39

51

10

7

25

7

2

1

1

1

956

439

439

12

12

14

14

34

34

12

12

430

30

92

25

184

29

18

32

5

15

15

15

1

1

1

1

216

216

80

6

1

55

18

35

11

24

30

30

20

20

51

18

33

15

15

15

15

17

17

17

17

11

11

11

11

63

63

63

63

236

236

17

17

14

14

53

17

17

19

85

33

18

34

1

1

66

48

18

323

27

27

27

172

172

172

124

124

124

1046

1046

1

1

40

40

3

1

1

1

17

17

33

33

867

25

372

470

85

69

16

7828

25

25

25

25

1

1

1

1

73

2

2

2

2

2

2

3

3

3

55

29

29

10

10

16

16

3

3

3

5

3

3

1

1

1

1

3

3

3

5212

1

1

1

78

78

78

38

38

38

162

162

162

2549

1315

1315

335

22

6

65

242

899

42

34

56

25

46

1

11

80

75

3

462

64

146

1

1

100

100

30

30

15

15

3

3

3

76

23

23

53

17

36

1

1

1

2061

12

12

10

10

13

13

100

100

30

30

119

99

20

89

81

8

642

150

492

8

8

106

106

52

52

880

880

88

88

88

9

9

9

51

29

29

28

1

22

22

22

22

22

21

1

20

1

1

21

21

21

21

21

21

21

21

56

56

56

56

2243

490

166

166

2

2

322

322

1753

27

27

34

34

14

4

10

1678

567

7

4

14

536

93

1

16

26

414

1

1

1

1

13

13

13

13

89

53

53

1

49

3

28

28

28

8

2

2

6

6

472

315

315

18

18

18

297

28

28

1

1

25

25

18

18

59

59

82

36

46

84

24

58

2

157

129

45

20

20

1

1

24

23

1

65

10

10

55

9

46

19

19

19

1

1

1

1

27

27

27

27

149

149

149

149

149

64

32

53

13636

10317

1

1

1

1

11

11

11

11

1

1

1

1

7

7

7

6

1

1537

1374

9

7

2

2

2

16

1

4

1

8

1

1

76

32

44

90

29

61

21

21

44

3

39

2

40

13

4

22

1

31

29

1

1

48

48

1

1

41

1

39

1

92

92

1

1

4

4

2

1

1

9

9

3

3

69

11

55

3

126

126

3

3

46

7

39

463

258

4

31

93

30

2

1

1

43

128

128

9

2

7

163

163

30

133

8475

4941

295

295

2900

90

2810

781

781

231

231

724

568

31

123

2

8

8

2

2

1664

1

1

22

22

1423

1423

177

177

41

41

1870

915

915

955

955

30

30

21

21

9

4

2

1

1

1

38

26

26

26

12

12

12

58

27

27

27

1

1

1

30

30

30

159

119

1

1

35

1

1

29

4

6

6

5

1

1

2

1

28

28

31

12

19

7

7

6

6

9

9

9

24

2

1

1

2

2

2

2

18

5

13

7

7

7

2814

947

281

13

13

178

178

58

58

2

2

30

30

666

666

3

134

2

2

7

385

48

1

45

1

4

16

18

28

28

8

8

20

20

256

112

43

43

14

4

9

1

46

46

6

4

2

3

3

144

52

47

5

27

27

19

19

3

3

14

1

13

6

6

11

11

2

2

9

3

6

1

1

7

7

7

2

5

630

630

458

28

112

201

4

2

2

35

1

32

1

32

8

44

44

44

44

84

54

30

92

92

2

2

90

3

87

1

1

1

1

718

8

8

6

2

1

1

1

158

148

31

4

69

32

5

2

5

10

8

2

2

2

2

547

1

1

1

1

8

2

4

2

3

1

2

289

91

96

1

101

4

4

93

92

1

13

13

109

1

108

2

2

12

12

12

2

10

2

2

2

130

130

130

126

4

5

5

5

5

27

27

27

27

27

459

459

94

94

78

11

5

365

20

20

345

4

192

149

19

19

19

19

19

134

43

22

18

18

18

4

4

4

17

17

17

17

4

2

2

2

2

2

2

1

1

1

1

1

34

5

5

5

5

15

15

15

15

1

1

1

1

7

7

7

7

6

3

3

3

3

3

3

56

56

56

56

56

6410

6378

1078

3

3

3

1020

1

1

48

48

43

43

10

1

9

295

21

6

252

16

197

113

69

15

15

15

21

14

7

72

72

164

61

103

154

154

55

55

55

24

15

1

1

14

14

9

9

9

4768

83

3

3

3

3

4

4

73

73

8

1

1

5

2

3

2

2

52

52

52

3

3

1

2

2965

2965

692

2273

40

40

1

11

17

1

3

7

292

3

3

275

275

14

6

3

3

1

1

1256

1

1

367

1

56

310

30

8

22

8

8

693

154

36

29

269

205

157

3

56

4

30

53

11

69

69

69

85

33

33

33

52

14

14

38

10

28

423

2

2

2

127

12

12

115

32

48

35

26

23

23

3

3

268

1

1

154

7

20

94

33

1

1

112

12

23

31

1

3

12

1

4

8

17

16

16

16

16

16

3

3

3

3

3

13

13

13

13

13

344

212

212

212

212

212

132

132

132

74

50

24

4

4

54

54

225

225

225

225

225

130

95

57

57

57

3

3

3

54

54

54

1

1

1

1

1

1

1
